# Blockchain Based Decentralized and Proactive Caching Strategy in Mobile Edge Computing Environment

**DOI:** 10.3390/s24072279

**Published:** 2024-04-03

**Authors:** Jingpan Bai, Silei Zhu, Houling Ji

**Affiliations:** School of Computer Science, Yangtze University, Jingzhou 434023, China; jingpan89@yangtzeu.edu.cn (J.B.); 2023710683@yangtzeu.edu.cn (S.Z.)

**Keywords:** caching, decentralization, mobile edge computing, blockchain, smart contract

## Abstract

In the mobile edge computing (MEC) environment, the edge caching can provide the timely data response service for the intelligent scenarios. However, due to the limited storage capacity of edge nodes and the malicious node behavior, the question of how to select the cached contents and realize the decentralized security data caching faces challenges. In this paper, a blockchain-based decentralized and proactive caching strategy is proposed in an MEC environment to address this problem. The novelty is that the blockchain was adopted in an MEC environment with a proactive caching strategy based on node utility, and the corresponding optimization problem was built. The blockchain was adopted to build a secure and reliable service environment. The employed methodology is that the optimal caching strategy was achieved based on the linear relaxation technology and the interior point method. Additionally, in a content caching system, there is a trade-off between cache space and node utility, and the caching strategy was proposed to solve this problem. There was also a trade-off between the consensus process delay of blockchain and the caching latency of content. An offline consensus authentication method was adopted to reduce the influence of the consensus process delay on the content caching. The key finding was that the proposed algorithm can reduce latency and can ensure the security data caching in an IoT environment. Finally, the simulation experiment showed that the proposed algorithm can achieve up to 49.32%, 43.11%, and 34.85% improvements on the cache hit rate, the average content response latency, and the average system utility, respectively, compared to the random content caching algorithm, and it achieved up to 9.67%, 8.11%, and 5.95% increases, successively, compared to the greedy content caching algorithm.

## 1. Introduction

Recently, mobile edge computing (MEC) has created a remarkable achievement for the cellular communication industry and has simplified humans’ lifestyle [1]. With the near user resources of computation and storage, MEC can provide a low-delay service for resource-constrained user terminals (UTs) on the internet of things (IoT) [2]. However, with the increase of the number of UTs and emerging smart applications, wireless communication networks face a serious challenge. The limited bandwidth and backhaul link become the bottleneck of network performance improvement. Thus, the question of how to reduce the traffic load of the communication network and data access delay becomes an urgent issue.

Edge caching is a very promising technology. It caches the requested data to nodes near UTs in advance to reduce the data delivery delay and the service cost. In the MEC environment, edge caching can provide a timely data response service for intelligent scenarios, such as intelligent transportation [3], intelligent manufacturing [4], intelligent security [5], smart grid, etc., so that the data access delay is effectively reduced. In this case, the data, which are frequently accessed or can be used repeatedly, can be downloaded to edge nodes. However, due to the limited storage capacity of edge nodes, the edge nodes cannot cache all data requested by UTs in advance. Thus, the question of how to reasonably select the cached data becomes a challenge. In addition, due to the self-deployment of edge nodes and the multiple data providers (DPs) coexisting, the traditional centralization transaction and auditing body limit the development of IoT. In the multiple DPs scenario, it is possible that there are malicious nodes that distort the cache data and broadcast the malicious contents. Thus, the question of how to realize the decentralized security data caching is also a challenge. Meanwhile, the existed caching strategies lack the incentive mechanism to encourage nodes to participate in the data caching. Therefore, with the advantages of decentralization, security, traceability, and automate management of blockchain (BC) and considering the content popularity, the content access delay, and the incentive mechanism, a node utility-based decentralized and proactive caching strategy was proposed in a mobile edge computing environment.

**Figure 1:**
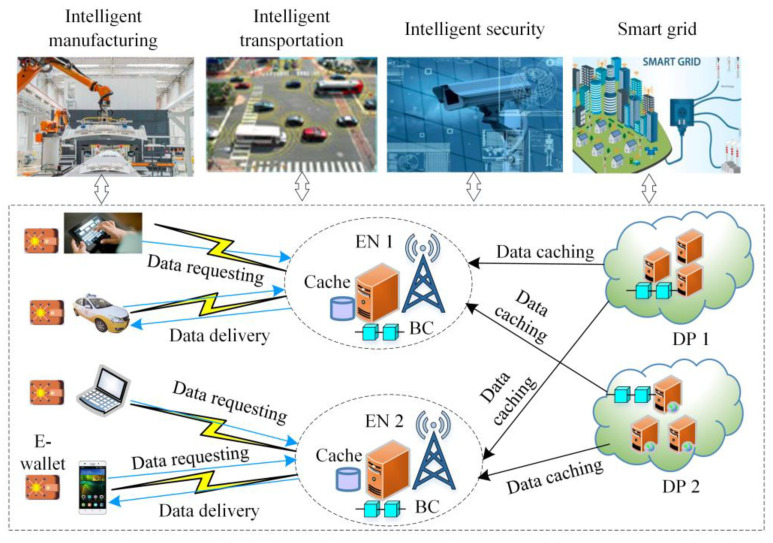
The content caching in mobile edge computing.

As shown in Figure 1, in the node utility-based decentralized and proactive caching strategy, the DPs release the smart contract for the requested contents. Then, by considering the content popularity, the content access delay, and the incentive mechanism, the node utility-based decentralized and proactive caching model was built, and the corresponding optimization problem was proposed. Furthermore, the linear relaxation technology and the interior point method were adopted to achieve the optimal caching strategy. Finally, the DPs release the smart contract to cache the selected contents and take the content caching and delivery as the transactions for storing into BC, so that the content requesting is traceable.

There is a trade-off between the cache space and the node utility. As the cache space capacity increases, edge nodes can cache more content. Thus, the node utility will rise. However, due to the limited cache space of edge nodes, the required contents must be selectively cached. Thus, there is a trade-off between the cache-space and the node utility. A proactive caching strategy was proposed to address the problem. Accordingly, the corresponding optimization problem was built. Furthermore, the linear relaxation technology and the interior point method were adopted to achieve the optimal caching strategy.

Additionally, there is a trade-off between the consensus process delay of the blockchain and the latency of content caching. The caching delay of content includes the consensus process delay of the blockchain and the content transmission delay from the cloud to edge nodes. The consensus process delay of the blockchain impacts the caching delay of nodes, and it is not suitable for the delay-sensitive IoT environment; thus, we adopted an offline way to reduce the impact of block generating on the content caching, i.e., after user terminals purchase the cache space, the data providers cache the contents in the cache space. Then, the blockchain consensus process is carried out. Certain security is sacrificed during the consensus process to achieve lower latency.

The main contributions and novelty are summarized as follows.

- The decentralized data caching system based on blockchain was proposed to trace back to the service and avoid the malicious behavior.
- The node utility-based decentralized and proactive caching optimization problem was built, the linear relaxation technology, and interior point method were adopted to achieve the optimal caching strategy.
- The simulation experiment environment was built. The results showed that the proposed algorithm can achieve better performance on the cache hit rate, the average content response delay, and the average system utility than that of benchmark algorithms.

The rest of this paper is organized as follows. Section 2 introduces the related works. Section 3 describes a decentralized data caching system based on blockchain, and Section 4 builds the proposed node utility-based decentralized and proactive caching optimization problem. In Section 5, the node utility-based decentralized and proactive caching algorithm is designed. Section 6 conducts the extensive experiments to verify the performance of the proposed algorithm. Finally, the conclusion is described in Section 7.

## 2. Related Work

Data caching is a promising technology used to reduce content transaction delay and cost. Currently, a large area of research exists on the data caching in academia and the industry. However, the research on data caching, comparing it with BC, is still in the beginning stage domestically and overseas. In this section, the related works are discussed. Then, the limitations of existing works are summarized. The summary of references is listed in Table 1.

In order to avoid the falsification of cached content and the malicious content broadcasting, Liu et al. proposed the BC-based data caching system for the vehicle edge computing network, in which a content index method, including the provider’s address and hash value of the content, is adopted to ensure the effectiveness of the content [6]. Meanwhile, the content index is stored into the blockchain to prevent tampering through the tamper resistance and distributed architecture of the blockchain. In order to encourage more ENs to share the storage resources and ensure the data reliability, Liu et al. designed an edge caching service architecture for caching transactions and content sharing [7]. For the mobility and low-latency requirement of Internet of Vehicles (IoV), Chai et al. proposed a two-layer BC-assisted active caching strategy to avoid the case that the conventional public blockchain systems suffer large consensus latency and cannot be well applied to IoV with the high mobility of vehicles and low latency requirement [8]. Xu et al. designed a new BC-based credible edge caching scheme, in which the transactions between ENs and UTs are supervised by a BC system with a decentralized method [9].

With the advantages of BC, Wang et al. proposed a decentralized active caching method for the hierarchical wireless network, in which the smart contracts are built to form the self-data caching market, and the self-content delivery is realized between incredible nodes [10]. In order to realize intelligent and secure data caching, Dai et al. built the decentralized IoV by combining deep reinforcement learning and the permission BC for the peer-to-peer transactions [11]. Sharma et al. proposed a neural BC-based ultrareliable caching for edge-enabled UAV networks, in which the BC is adopted to ensure the ultra-reliability communication and to form a flat architecture, and the neural model fortifies an efficient transport mechanism [12]. Guo et al. designed a decentralized and creditable authenticator system based on the BC and MEC. Furthermore, based on this system, a corresponding caching strategy was proposed to improve the cache hit rate [13]. M. Burhan et al. examined the layered architecture of fog-based IoT networks alongside IoT applications operating within the context of the fog computing paradigm [14]. Faheem M et al. proposed a blockchain-based smart contract framework in Solana blockchain for integrating and monitoring distributed energy resources (DERs) in the smart grid. The framework, called advanced Solana blockchain (ASB), enables secure and resilient real-time control and monitoring of DERs [15]. Malik H et al. reviewed the integration of blockchain technology (BCT) and internet of things (IoT) in drug supply management (DSM) and smart cities (SC), categorizing research articles and identifying motives for their use. It offers recommendations for future research, highlighting opportunities for creating decentralized DSM and SC applications [16]. Raza B et al. proposed a cluster-based autonomic performance prediction framework using a case-based reasoning approach to enhance data warehouse performance [17].

In conclusion, fewer existing works have considered the relationship between the limited cache capacity and strict delay requirements, as well as the resource sharing willingness when designing caching strategies. The existed works mainly focus on the content preference, node adjacency user association, and quality of experience. Although the caching decision can be achieved based on those factors considered by the existing works, more nodes participating in the resource sharing will improve the performance of content caching. Thus, the incentive mechanism is very important for edge caching. In this paper, node utility was considered to build the active data caching strategy with the limitation of content delivery delay.

## 3. The Decentralized Data Caching System Based on Blockchain

**Figure 2:**
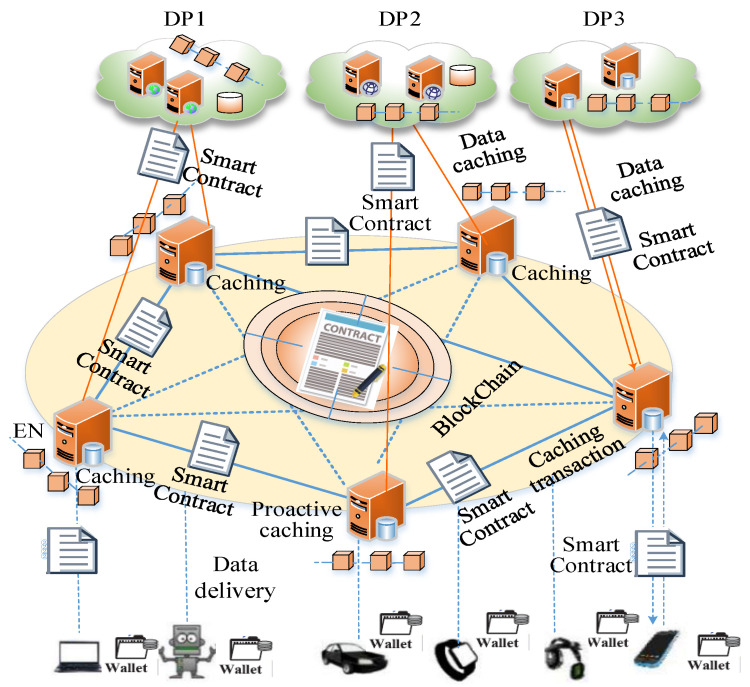
The decentralized data caching system based on blockchain.

In the edge intelligence scenario, the decentralized data caching system based on blockchain (DDCSBC) consists of data providers (DPs), edge nodes (ENs), user terminals (UTs), and the blockchain (BC) network, which is shown in Figure 2.

The DPs provide the data for the UTs requiring data. The data include the videos, audio, text, figures, neural network model, etc. Each DP denotes the independent entity, such as NetFlix, or the federation between the independent entity and the telecom provider, such as Orange, Akamai [18], etc. Each user represents the UT who buys or consumes the contents or services from DPs. ENs consist of the edge devices with distributed deployment, which provide the data delivery service for UTs. The BC network is taken as the core entity of management to provide the decentralized safety data management service for data caching. The BC network consists of the P2P network of ENs and is used to conduct transactions, verify blocks, and append the new block into the blockchain.

It is assumed that all of the entities who participated in the DDCSBC finished the identity register of the BC network. The identity register of each DP or UT is finished by the transactions between blockchain nodes. The subscription of data caching is conducted between UTs and DPs based on the smart contract [19,20,21]. Before the data is cached, Each UT must submit its subscription to the DPs. The smart contract is used for content delivery. Content delivery includes two stages, i.e., the content prefetching and the content delivery execution. Each stage is conducted with the corresponding smart contract.

### 3.1. The Content Prefetching

**Figure 3:**
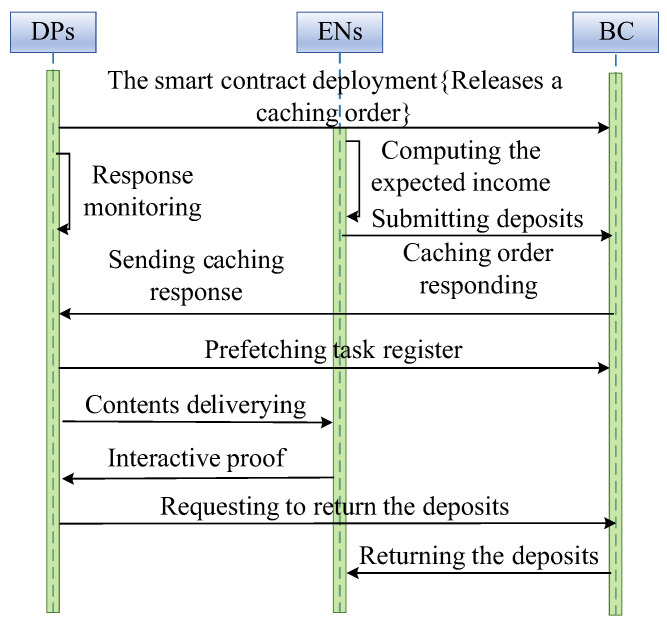
The content prefetching process.

In the content prefetching stage, DPs and ENs negotiate for the cached contents as shown in Figure 3. The detailed process for the content prefetching is described as follows.

- The DP k releases a caching order for each content q∈F, in which the caching order is released by the corresponding smart contract and is used to make a price $o_{n,k}$ for the content delivery.
- EN m will determine whether to cache the content q by computing the expected income. If EN m wants to cache content q, it will call cache providing the function to respond DP k. Meanwhile, EN m sends the deposits to the corresponding smart contract.
- The smart contract will send a response to DP k for the EN’s response. In this paper, it is assumed that one DP can choose multiple ENs for data caching.
- The DP k identifies the selected EN m by calling the register function of the smart contract. Then, DP k transmits the copies of content q to EN m by the third-party methods.
- In order to achieve the deposits, the EN m must provide the interactive proof [22] of content q for DP k, so that the cached contents can be retrieved.
- After the interactive proof is verified, the DP k will trigger the smart contract to return the deposits to EN m.

When the content prefetching stage is finished, the smart contracts for content prefetching will be destroyed so that the unfinished transactions do not exist. Meanwhile, a group of new smart contracts will be deployed.

### 3.2. The Content Delivery Execution

**Figure 4:**
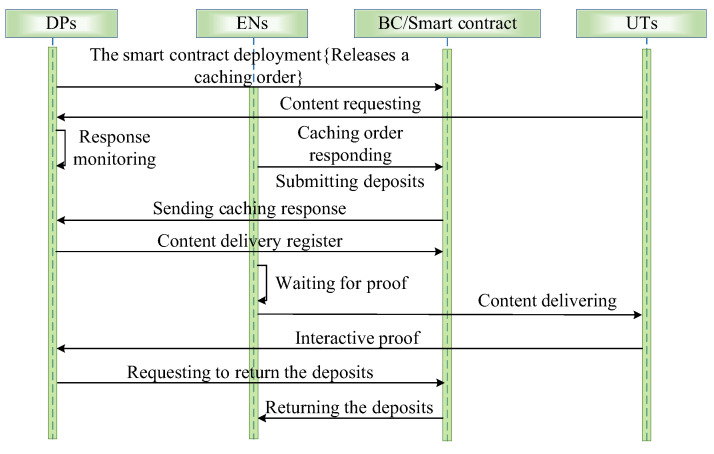
The sequence diagram of the smart contract.

In the content delivery execution stage, each EN send the cached contents to UTs by the smart contract of content delivery. The smart contract of content delivery is shown in Figure 4. The detailed process for the content delivery execution is described as follows.

- The DP k releases a delivery order for each content q∈F by the corresponding smart contract. The smart contract is regarded as the escrow account so that each DP k pays for the delivery order until the corresponding content delivery is finished.
- The EN m responds to the content delivery order by calling the content delivery function. Meanwhile, it sends the deposits to the smart contract of content delivery.
- The smart contract of content delivery triggers one event to inform DP k about the response of EN m. In this paper, it is assumed that one DP can choose multiple ENs for content delivery.
- The DP k allocates the content delivery tasks to the ENs who respond to DP k and registers the content delivery task for each corresponding EN m by the smart contract of content delivery. In addition, the deposits $o_{k,q}$ of DP k for the delivery of content q are also held by the corresponding smart contract until the corresponding UTs provide the interactive proof of content q for DP k. In order to prevent ENs and UTs from cheating the DPs for rewards without the content delivery, the smart contract of content delivery demands that UTs provide the interactive proof within a special delay for certificating the content delivery. Otherwise, the smart contract of content delivery will roll back, and the DP k will receive the deposits as the discipline of EN m.
- After the interactive proof of content q is verified successfully, DP k will return the deposits to EN m by the smart contract of content delivery.

When the content delivery execution stage is finished, the smart contract of content delivery will be updated, and the unfinished transactions will be destroyed.

### 3.3. The Description of Smart Contract

The smart contract is actually an agreement, which is an automated contract system based on blockchain technology and runs on the top of blockchain. Visually speaking, the smart contract digitalizes the contract item to be executed in daily life. The smart contract ensures certain security of the system. The reason is that if an event triggers an item in the contract, the smart contract will automatically execute the agreement between the involved parties. The smart contract enables decentralized automation by enforcing and validating the terms of the multi-party agreement, which promotes the efficiency of the system [23].

**Figure 5:**
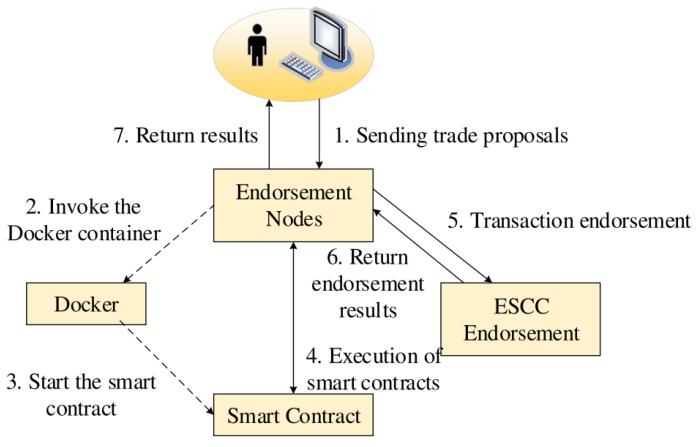
The workflow of smart contracts [24].

Thus, smart contracts do not require a third party to monitor the execution of the contract. If the environment for the contract execution can be guaranteed to be trustworthy, the contract will be executed automatically. In addition, smart contracts are codes on the top of the blockchain, which make it so the corresponding transaction records cannot be tampered with when the contract is signed. Smart contracts also require both parties to provide a certain cost to prevent malicious trading, ensuring the normal execution of transactions. The service provider will only provide the service when the user pays a deposit from their account to the contract. If one party maliciously breaches the contract, then the defaulting party will be punished for asset losses. Therefore, smart contracts can effectively prevent malicious transactions. The specific working flowchart of smart contracts is depicted in Figure 5.

### 3.4. The Analysis of the System Security

In this section, we discuss the security of the system. Then, we discuss the security, credibility, traceability, integration, and integrity of the system to analyze the security, respectively.

- The security of the system

In the blockchain network, entities need to register or authenticate their identities before joining the blockchain network, and they interact with information in the blockchain network in an anonymous manner. For example, the transaction requester uses the public key as a pseudonym, thus guaranteeing the anonymity of the real identity. The transaction information in the blockchain is signed, and only the node with the correct private key can access the transaction. If a malicious node wants to authenticate a transaction, it must forge the private keys of other nodes associated with the transaction. However, the malicious node only has the public key information of other nodes, and there is no feasible way to obtain the corresponding private key from the public key; thus, the malicious node cannot implement the forging of the private key.

In the proposed algorithm, content caching and space provision can be authenticated and recorded in the blockchain as transaction data to ensure their security.

- The credibility of the system

In the process of cache content request and cache space provision, if the relevant transaction requires the participation of a trusted third party, the security of the system largely depends on the security of the third party. If the security of the third party cannot be guaranteed, the contents of the system will be exposed to greater risks.

In the proposed algorithm, the blockchain establishes trust between physical nodes through smart contracts, avoiding the participation of third-party entities to achieve mutual trust node interaction, and at the same time, it also improves the robustness and scalability of the system.

- The traceability of the system

In the proposed algorithm, all broadcast transaction information is permanently recorded by the whole node of the blockchain and is time-stamped. At the same time, these transactions cannot be modified by a single node. Since the blockchain is a distributed ledger, transactions are updated synchronously and can be easily obtained from any full node. When malicious behavior occurs, any node can easily verify and track previous records by accessing the full node. The timestamp in the blockchain guarantees the integrity of the transaction and prevents the transaction information from being forged or tampered with. The smart contract runs on the top of the blockchain, which is a self-executing contract with terms of the agreement between involved parties. The smart contract allows for decentralized automation by enforcing and verifying the conditions of the multiparty agreement. By using smart contract technology to support the distributed services, the system can be more effective and efficient without any intermediaries.

- The integration of the system

In a blockchain system, the consensus process requires a lot of caching content resources and is characterized by high latency. Thus, the combination of blockchain with the layered architecture of mobile edge computing has led to a significant rise in latency in the content delivery process. To solve this problem, this paper recorded the caching process of contents in an offline way, which provides resources first and then records them to the blockchain to reduce the impact of the delay generated by blocks on the caching delay of contents. In the above way, the impact of the high latency characteristics of the blockchain on the content caching process is reduced, allowing the blockchain and the hierarchical architecture of mobile edge computing to achieve better integration.

- The integrity of the system

Each transaction is transmitted to all nodes in the blockchain network through broadcasting. Unauthenticated transactions are temporarily stored in the transaction pool of all nodes. When the transaction volume reaches a certain threshold or the transaction waiting time exceeds a certain threshold, the outgoing node will package the transactions in the transaction pool and generate new blocks. After the new block passes the candidate node authentication, it will be broadcasted across the entire network. All nodes will add new blocks to their own blockchain, while light nodes will add new block headers to their own blockchain, allowing all nodes to fully record resource requests, provision, and other related data.

## 4. The Node Utility-Based Decentralized and Proactive Caching Strategy

**Figure 6:**
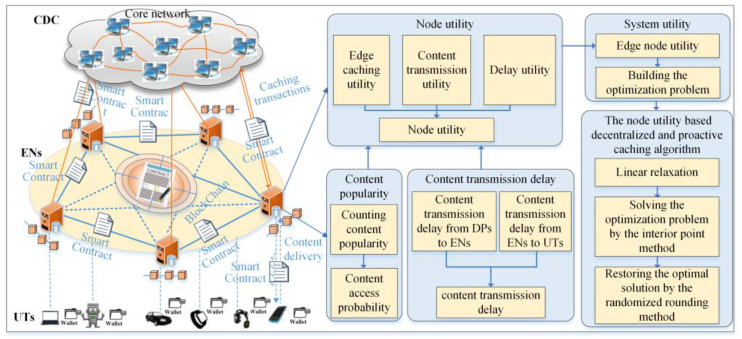
The overview of the NUDPC strategy.

The node utility-based decentralized and proactive caching (NUDPC) strategy is described in Figure 6. Firstly, the ENs inquire about the access times of contents in recent days by the BC, and they compute the content popularity. Then, the data transmission rate between the cloud data center (CDC) and ENs, between ENs and UTs, is computed, successively, so that the content delivery delay is achieved. Moreover, the content delivery delay and the node utility are considered to build the caching optimization problem. Finally, the linear relaxation technology, the interior point method, and the randomized rounding technology are adopted to achieve the optimal strategy of data caching. The interaction between nodes is conducted with the smart contract in the DDCSBC so that the security of data processing is guaranteed. For convenience, the major notations used in this article are summarized in the Abbreviation.

As shown in Figure 6, the set of ENs is denoted by $BS=\left\{1,2,\ldots ,M\right\}$. The available cache storage size is $C_{m}$. The set of DPs is represented by $DP=\left\{1,2,\ldots ,K\right\}$. Let $U_{m}=\left\{1,2,\ldots ,N_{m}\right\}$ be the set of UTs served by m-th EN. Each UT communicates with the EN by the wireless link, and the ENs communicate with the CDC by the optical fiber. In this paper, the content caching strategy for a single slot was studied. In the single slot, the location of the nodes is unchanged. The set of contents is denoted by $F=\left\{1,2,\ldots ,Q\right\}$, and the size of q-th content is $D_{q}$. It is assumed that the content popularity follows as the Zipf distributed function. Then, the popularity of the q-th content is represented by the following:

(1) $$p_{q}=\frac{1/q^{\eta }}{\sum_{\alpha =1}^{Q}1/\alpha^{\eta }},\;0<\eta <1,$$

where α denotes the rank of content requesting times, and η is a positive constant. The larger η is, the larger the reuse rate is. Moreover, the most popular content accounts for the majority of download requests [25].

### 4.1. The Communication Model

Let $R_{k,m}$ be the data transmission rate between DP k and EN m, and $R_{m,n}$ denotes the data transmission rate between EN m and UT n ($n\in U_{m}$). Then, $R_{m,n}$ is denoted by the following:

(2) $$R_{m,n}=W_{m,n}\log\left(1+\frac{P_{m,n}|G_{m,n}{|}^{2}}{\sigma_{m,n}^{2}}\right),\forall m\in BS,\forall n\in U_{m},$$

where $W_{m,n}$ is the bandwidth, which is allocated to UT n by EN m, and $P_{m,n}$ is the transmission power of EN m. Meanwhile, $|G_{m,n}{|}^{2}$ denotes the channel gain between EN m and UT n, and $\sigma_{m,n}^{2}$ is the Gaussian white noise.

### 4.2. The Content Transmission Delay Model

When the UTs request the contents, the corresponding EN transmits the contents to these UTs if the requested contents are cached into the EN. Otherwise, the contents are transmitted to these UTs from DPs. In the single slot, each DP releases the smart contract of content prefetching, and each EN selects the cached contents according to its utility. Let $x_{m,q}$ be the decision variable of content caching. $x_{m,q}=1$ if EN m decides to cache the q-th content. Otherwise, $x_{m,q}=0$.

Thus, if EN m has cached the q-th content, then the content transmission delay for content q from EN m to UT n is denoted by the following:

(3) $$t_{m,n}^{q}=\frac{D_{q}}{R_{m,n}}$$

where $D_{q}$ denotes the size of content q.

If EN m has not cached the q-th content, then the content transmission delay for content q from DP k to UT n is represented by the following:

(4) $$t_{k,n}^{q}=\frac{D_{q}}{R_{k,m}}+\frac{D_{q}}{R_{m,n}},$$

Then, the content transmission delay of content q achieved by UT n is as follows:

(5) $$t_{n}^{q}=\left(1-x_{m,q}\right)\underset{k\in DP}{\min}t_{k,n}^{q}+x_{m,q}t_{m,n}^{q}.$$

### 4.3. The Content Preference Model

Usually, the different UTs have different content preferences due to the different preference or charging. Thus, let $\Theta_{n}=\left\{\theta_{n,1},\ldots ,\theta_{n,q},\ldots ,\theta_{n,Q}\right\}$ be the different preference of UT n, where $\theta_{n,q}$ follows as the Zipf distributed function:

(6) $$\theta_{n,q}=\frac{1/\rho_{n,q}^{\gamma }}{\sum_{\delta_{n,q}=1}^{Q}1/\delta_{n,q}^{\gamma }},q\in F,n\in U_{m},$$

where $\rho_{n,q}^{\gamma }$ denotes the preference rank of content q for UT n, and γ is a positive constant. γ denotes the preference distribution of UTs in the content [26].

### 4.4. The Node Utility Model

In the actual application scenario, node utility consists of edge caching utility, content transmission utility, and delay utility. The detailed description of node utility is shown as follows.

Due to the limited storage capacity of ENs, the caching service provided by ENs is not free. The DPs should pay for the caching service of ENs. In addition, content caching will consume the energy of ENs, and the energy consumption is related to the cached content size. Thus, each EN should decide the caching price. The edge caching utility denotes the profit achieved by ENs with the caching storage renting, which can be represented by the following:

(7) $$prof_{m}^{1}=\sum_{q=1}^{Q}\left(g^{cache}-g^{cost}\right)\cdot x_{m,q}\cdot p_{q}\cdot D_{q},$$

where $g^{cache}$ is the price of unit caching storage space, and $g^{cost}$ denotes the maintenance cost of unit caching storage space. Moreover, $\sum_{q=1}^{Q}x_{m,q}\cdot p_{q}\cdot D_{q}$ represents the storage size used by the cached contents, and $\sum_{q=1}^{Q}x_{m,q}\cdot p_{q}\cdot D_{q}\leq C_{m}$.

If the ENs cache the contents required by UTs, then the contents will be delivered to the UTs. In this case, there is no transmission delay between DPs and ENs, and the backlink bandwidth is consumed, and the content transmission costs are reduced. Thus, the content transmission utility is defined as follows:

(8) $$prof_{m}^{2}=g^{backhaul}\cdot \sum_{q=1}^{Q}x_{m,q}\cdot p_{q}\cdot D_{q},$$

where $g^{backhaul}$ denotes the profit per unit backlink bandwidth saved by UTs.

In the edge intelligence environment, the reward-punishment mechanism (RPM) of content delivery was designed to reduce the content transmission delay and improve the quality of service (QoS). Specially, if the required contents are achieved by the UTs before deadline time, then the ENs will obtain the corresponding reward. Obviously, the less content transmission delay, the bigger the reward. If the content transmission time is more than the deadline time, then the ENs will be punished according to the length of overtime. If the deadline time of UT n for content requirement is $t_{n}^{0}$, then the delay utility of EN m is defined by the following:

(9) $$prof_{m}^{3}=\sum_{n=1}^{N_{m}}\sum_{q=1}^{Q}g^{time}\cdot \theta_{n,q}\cdot \Delta t_{n}^{q},$$

where $\Delta t_{n}^{q}=t_{n}^{0}-t_{n}^{q}$ denotes the length of advanced time that EN m transmits the content q to UT n. $g^{time}$ is the reward per unit advanced time for EN m.

Thus, the node utility is shown as follows:

(10) $$prof_{m}=prof_{m}^{1}+prof_{m}^{2}+prof_{m}^{3}.$$

The aim of the NUDPC strategy is to maximize the system utility with the limit of content transmission delay. Thus, the optimization problem of the NUDPC is as follows:

(11) $$P1:\;\max\sum_{m=1}^{M}prof_{m},$$

*s*.*t*.

(12) $$\sum_{q=1}^{Q}x_{m,q}p_{q}D_{q}\leq C_{m},\forall m\in BS,$$

(13) $$x_{m,q}\in \left\{0,1\right\},\forall m\in BS,\forall q\in F,$$

where the constraint (12) ensures that the size of the cached contents cannot be more than the storage space of each EN, and the constraint (13) defines the decision variables. Obviously, the optimization problem of the NUDPC is the 0–1 integer linear programming (ILP) problem, which belongs to the NP-hard problem [27].

## 5. The Node Utility-Based Decentralized and Proactive Caching Algorithm

In order to achieve the optimal solution of problem P1, the discrete variables are relaxed to [0, 1], i.e., optimization problem P1 is converted to the optimization problem P2 as follows:

(14) $$P2:\;\max\sum_{m=1}^{M}prof_{m},$$

*s.t.*

(12),

(15) $$x_{m,q}\in \left\{0,1\right\},\forall m\in BS,\forall q\in F.$$

Obviously, the optimization problem P2 is the linear programming (LP) problem, which can be solved with the interior point method. Firstly, the optimization problem P2 is converted into the unconstrained optimization problem. Then, the optimal solution is obtained based on Newton’s method.

The optimization problem P2 is converted to the standard linear programming problem as follows:

(16) $$P3:\;\min f_{0}\left(x\right)=-\sum_{m=1}^{M}prof_{m},$$

*s.t.*

(12), (15).

Then, the penalty function is defined by the following:

(17) $$\min f_{1}\left(x,\lambda \right)=f_{0}\left(x\right)+\lambda \varphi \left(x\right),$$

where λ is the penalty factor, and $\varphi \left(x\right)$ is the barrier function whose form is shown as follows:

(18) $$\varphi \left(x\right)=-\sum_{m=1}^{M}\log\left(C_{m}-\sum_{q=1}^{Q}x_{m,q}p_{q}D_{q}\right)-\sum_{m=1}^{M}\log x_{m,q}-\sum_{m=1}^{M}\log\left(1-x_{m,q}\right).$$

Furthermore, the newton iterative equation is shown as follows:

(19) $$x_{m,q}^{\left(k+1\right)}=x_{m,q}^{\left(k\right)}-H^{-1}\left(x_{m,q}^{\left(k\right)},\lambda \right)\cdot \nabla f_{1}\left(x_{m,q}^{\left(k\right)},\lambda \right),n\geq 0,$$

where k is the iteration time.

Thus, the pseudo code of the interior point method is described in Algorithm 1. Firstly, the initial values of the parameters are given. Then, the penalty function is built, and the optimal solution of optimization problem P2 is achieved (Algorithm 1 Line 2~3). Furthermore, Algorithm 1 stops if the stop condition is satisfied (Algorithm 1 Line 4~5). Otherwise, Algorithm 1 continually works (Algorithm 1 Line 6~8). Finally, the optimal solution $x^{*}$ is achieved (Algorithm 1 Line 11).
**Algorithm** **1:** The interior point algorithm for optimization problem P2**Input:** The initial value of penalty factor $\lambda^{\left(0\right)}$. The threshold of accuracy ε. The parameter C. The initial solution of optimization problem P2 $x^{\left(0\right)}$. The maximal iteration times $j_{\max}$. The initial iteration variable j.**Output:** The optimal solution of optimization problem P2 $x^{*}$.**While** $j\leq j_{\max}$.   Building the penalty function based on Equation (17).   $x^{*}(j+1)\leftarrow x^{*}(j)$ \\ Updating the optimal solution by Equation (19)    **If** $\left\|x^{*}(j+1)-x^{*}(j)\right\|\leq \varepsilon$     **Break**.   
**Else**
     $\lambda^{\left(k+1\right)}\leftarrow C\lambda^{\left(k\right)},C>0$.     j←j+1.   
**End If**
 
**End While**
 
**return** $x^{*}$


The node utility-based decentralized and proactive caching algorithm, which is described in Algorithm 2, includes three stages. In the first stage, the discrete variables of optimization problem P1 are relaxed, and the new optimization problem is achieved (Algorithm 2 Line 1). In the second stage, the optimal solution of the new optimization problem is obtained by the inter-point method (Algorithm 2 Line 2).

In the third stage, the optimal solution is restored by the randomized rounding method (Algorithm 2 Line 3~7), and the content caching strategy and the system utility are achieved (Algorithm 2 Line 8~9). The randomized rounding method [28] is described as follows:

(20) $$P\left[x=1\right]=x^{*},$$

where $P\left[x=1\right]$ denotes the probability of the variable x being 1, and $x^{*}$ is the optimal solution of optimization problem P2.

The time complexity of Algorithm 2 consists of the time complexity of Algorithm 1 and the time complexity of the randomized rounding method. The time complexity of Algorithm 1 is $O\left({\left(MQ\right)}^{3.5}\right)$ [29], where M is the number of ENs, and Q is the number of contents. The time complexity of the randomized rounding method is $O\left(MQ\right)$. Thus, the time complexity of Algorithm 2 is $O\left({\left(MQ\right)}^{3.5}\right)$.
**Algorithm** **2:** The node utility-based decentralized and proactive caching algorithm**Input:** The number of ENs M. The storage space $C_{m}$ of EN m. The number of UTs $N_{m}$ of EN m. The deadline time of UT n for content requirement is $t_{n}^{0}$. The content set F. The set of content size D.**Output:** The content caching strategy $x^{*}$ and the system utility $f\left(x^{*}\right)$. 1: The optimization problem P1 is converted to the optimization problem P2 by relaxing the discrete variables in the optimization problem P1.2: The optimal solution $x^{*}=\{{\tilde{x}}_{m,q}^{*}\}$ is achieved by solving the optimization P2 based on Algorithm 1.3: **For each** m∈BS **do**
4:  **For each**
q∈F
**do**5:   $P\left[x_{m,q}=1\right]\leftarrow {\tilde{x}}_{m,q}^{*}$.6:  **End For**7: **End For**8: $f\left(x^{*}\right)\leftarrow \sum_{m=1}^{M}prof_{m}\left({\tilde{x}}_{m,q}^{*}\right)$.9: **return**
$x^{*}$, $f\left(x^{*}\right)$


## 6. Simulation Experiments

### 6.1. Experimental Environment

**Figure 7:**
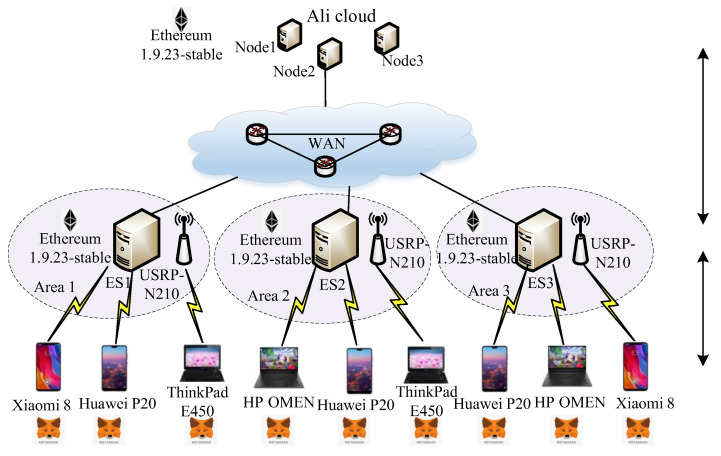
The experimental environment.

As shown in Figure 7, the experiment environment for the DDCSBC includes three Ali cloud servers, three Lenovo servers, multiple telephones, and lap computers. The Ali cloud servers are taken as the DPs and the company renting Ali Cloud servers for the experiment is Alibaba Cloud Company in Hangzhou, China. The Lenovo servers are regarded as the ENs and are distributed in different areas to form the decentralized Ens and the company is Lenovo Group in Beijing, China.. Meanwhile, these Ens are also the mining nodes for the BC system. The telephones and the lap computers are the UTs for sending content requests. The company of UTs including Huawei P20, Xiaomi 8, ThinkPad E450, HP OMEN are Huawei Group in Shenzhen, China, Xiaomi Group in Beijing, China, Lenovo Group in Beijing, China, HP Group in Palo Alto, CA, USA, respectively. The node configuration information on hardware is shown in Table 2. The node configuration information on the software is shown in Table 3.

### 6.2. Test Dataset

In order to verify the performance of the proposed algorithm, the MovieLens dataset [30] was taken as the test dataset. The MovieLens dataset is an open-source dataset and was published by the GroupLens team in the University of Minnesota System. This dataset includes the user ID, movie ID, comments on movies, timestamp for comments, etc. This dataset consists of 27,753,444 comments of 283,228 users on 58,098 movies from 9 January 1995 to 26 September 2018. In the experiments, the comments were taken as the history data of content requests. Specifically, the dataset generated from 1 January 2010 to 17 October 2016 was selected. For each user, the comments for one day were taken as the content requests for one minute to form the time series data of content requests [31].

### 6.3. Benchmark Algorithms

In order to verify the performance of the proposed algorithm, the random content caching (RCC) [32] and the greedy content caching (GCC) [32] were taken as the benchmark algorithms. In the RCC algorithm, the contents were randomly selected for caching. The RCC algorithm is the traditional caching algorithm and is usually taken as the benchmark algorithm. In the GCC algorithm, the contents with the most popularity are cached. The GCC algorithm is a common caching algorithm. Thus, it is reasonable to take the RCC algorithm and the GCC algorithm as the benchmark algorithms.

### 6.4. Metrics

The experiments included the cache hit rate (CHR), average content response delay (ACRD), and average system utility (ASU). The CHR is the ratio of the number of requested contents cached on ENs to the number of cached contents on ENs. The ACRD is the average time quantum from the acquirement of content requesting instructions on ENs to the acquirement of requested contents on UTs. The ASU is the ratio of the system utility to the number of UTs.

### 6.5. Experimental Results

In the experiments, each EN sends the requests of accessing the transaction history data to the BC system. If the address and signature of this EN are successfully verified, then the BC system will send the history data list to the EN. This EN computes the content requesting times and predicts the content popularity, respectively. Then, the node utility can be achieved based on the content popularity and the size of the contents. Furthermore, the cached contents are selected by Algorithm 2. Finally, this EN caches the selected contents in advance. The special value of the experiment parameters was set as follows.

The number of contents was set to 1000. The content size followed the uniform distribution with [10, 50] MB. The maximum tolerated delay of each request followed the uniform distribution with [5, 10] s. The cache space of each EN followed the uniform distribution with [5, 50] GB. The bandwidth between the cloud and ENs was 100 Mbps, and the bandwidth between ENs and UTs was set to 20 MHz. In addition, it was assumed that $g^{cache}=g^{backhaul}=g^{time}=1$, and $g^{cost}=0.5$. According to [11] and the values of Zipf parameter in [13], the parameters used in the simulation are listed in Table 4.

Furthermore, in order to verify the performance of the proposed algorithm, the influence of the Zipf parameter, the number of contents, the cache space, and the number of UTs on the metrics were discussed, successively. In each group of experiments, the experiment was conducted repeatedly 20 times, and the average value was taken as the experiment result.

#### 6.5.1. The Influence of the Zipf Parameter on the Metrics

In order to verify the influence of the Zipf parameter on the metrics, the Zipf parameter was set to 0.1, 0.2, 0.3, 0.4, 0.5, 0.6, 0.7, 0.8, 0.9, and 1, respectively. In addition, the number of UTs was set to 20, and the cache space of each EN was set to 20 GB.

**Figure 8:**
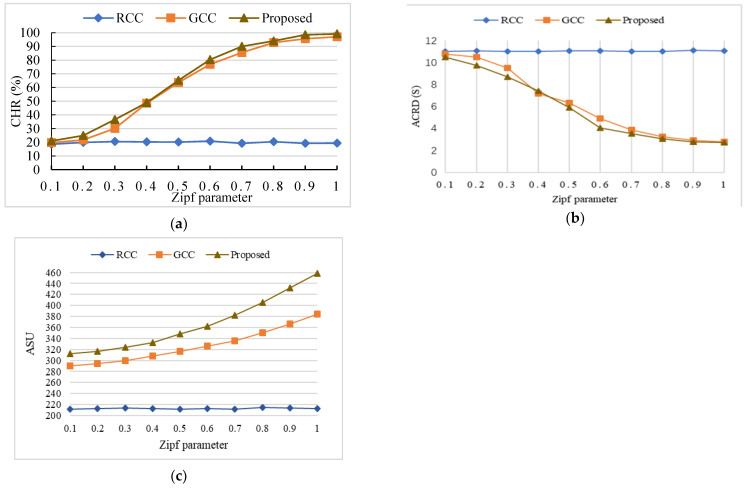
The influence of the Zipf parameter on the metrics: (**a**) the influence of the Zipf parameter on CHR; (**b**) the influence of the Zipf parameter on ACRD; (**c**) the influence of the Zipf parameter on ASU.

Figure 8 shows the influence of the Zipf parameter on the metrics. Each group of experiments was repeated 20 times under the same conditions, and the average value was used as the final experimental result. The results demonstrate that the proposed algorithm can obtain a higher performance in terms of CHR, ACRD, and ASU.

Figure 8 demonstrates that the content popularity was more concentratedly distributed as the Zipf parameter became large. This further led to an increase in CHR and ASU and a decrease in ACRD. From Figure 8a, we know that the value of CHR became larger with the increase of the Zipf parameter. This is because the content popularity was more concentratedly distributed as the Zipf parameter became larger, Then, the replicas of the popular content in the cache space of ENs can satisfy more requests. In this case, the CHR is improved. From Figure 8b,c, the ACRD of ENs reduced, and the ASU of ENs kept increasing when the parameter of the Zipf distribution rose. Since the proposed algorithm concurrently considers the content popularity and the size of contents for the cache decision, the ENs can satisfy more requests of UTs. In this case, the ACRD was reduced. In addition, the content of the UT request was cached on the EN in advance so that the UT could obtain the required content from the EN without generating the content transmission delay from the cloud to the EN, thereby improving the content transmission utility.

In the RCC algorithm, content popularity was not considered when selecting cache contents. In the GCC algorithm, when the cache contents were selected, the content popularity was considered, but the influence of the size of the contents on the storage space of ENs was neglected. In the proposed algorithm, the content popularity and the size of the contents were considered concurrently to select the cached contents, which promote that ENs can satisfy more requests from UTs. In addition, the incentive methods in the proposed algorithm were adopted, and the content response delay and the node utility were balanced. Thus, the proposed algorithm can achieve better performance on the metrics than the benchmark algorithms.

The cost caused by the Zipf parameter will increase with the decreased value of the Zipf parameter. This is because the content will be more dispersed as the value of the Zipf parameter decreases, which cannot satisfy the demands of most user terminals. Thus, the CHR decreases. Accordingly, the cost grows during the content caching process.

For example, when the Zipf parameter was set to 0.6, the proposed algorithm achieved 285.09% and 4.35% increases in CHR compared to the RCC algorithm and the GCC algorithm, respectively. The ACRD of the proposed algorithm was reduced up to 63.32% and 17.24% compared to that of the RCC algorithm and the GCC algorithm, successively, and the proposed algorithm achieved 69.95% and 11.04% improvements in ASU compared to the RCC algorithm and the GCC algorithm, respectively.

#### 6.5.2. The Influence of Contents on Metrics

**Figure 9:**
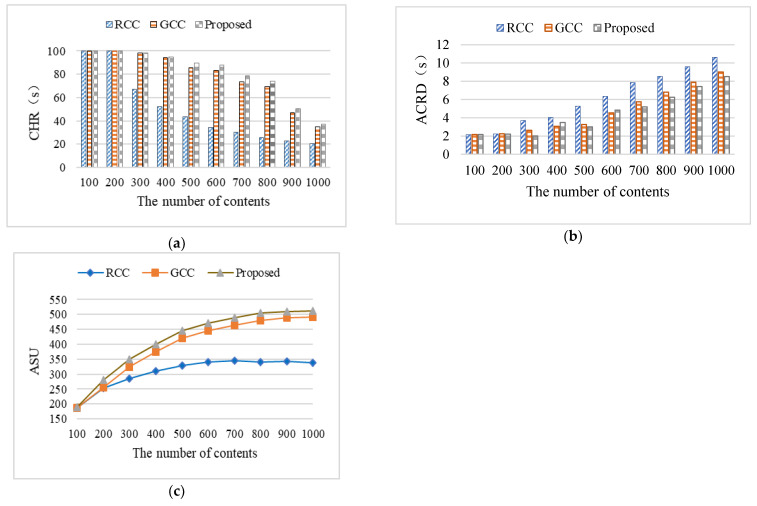
The influence of the number of contents on the metrics: (**a**) the influence of the number of con-tents on CHR; (**b**) the influence of the number of contents on ACRD; (**c**) the influence of the number of contents on ASU.

In order to verify the influence of the number of contents on metrics, the Zipf parameter was set to 0.6, the number of UTs was set to 20, the cache size of ENs was set to 10G, and the size of contents was set to 50 MB. The experiment was divided into 10 groups, with the content quantity for each group set to 100, 200, 300, 400, 500, 600, 700, 800, 900, and 1000, respectively. Figure 9 shows the influence of the number of contents on metrics. Each group of experiments was repeated 20 times under the same conditions, and the average value was used as the final experimental result.

The number of contents determines whether more content is cached on the edge nodes. With the increase in the number of contents, the CHR decreased and ASU and ACRD rose. Figure 9a shows the change of CHR with different sizes of content quantity. Within a single time slot, the CHR was close to 1 before the content quantity reached 200. After 200, the CHR decreased with the increase of content quantity in the content library. This is because when the number of contents is small, all contents can be cached to the local ENs, which can meet almost all terminal requests. While the number of contents increases, some contents cannot be cached to ENs, which own the limited edge cache space, and the local cache can only meet the demands of some terminals. This will lead to a decrease in CHR.

At the same time, when contents are cached on the local ENs, the content delivery delay becomes the transmission delay from the ENs to the UTs and the content delivery delay is small. The requested contents by UTs need to be obtained from the cloud. Thus, the content delivery delay increases. Figure 9b shows that when the number of contents was 200, the ACRD of the three caching algorithms was almost the same, at a low level. However, when the number of contents exceeded 200, the ACRD increased with the increase of the number of contents. The reason was that, with the increase of the number of contents, the edge caching space was limited; thus, more and more contents cannot be cached at the edge. When there are UT requests, more contents need to be obtained from clouds, which increases the content delivery delay. Under the same number of contents, the proposed algorithm had the shortest ACRD.

Figure 9c depicts how the value of ASU increased and finally stabilized as the number of contents grew. This is because, as the number of contents increased, more cache space was required, and the ENs could achieve more utility by renting out cache space. In addition, as more contents can be cached locally in the ENs, the utility obtained by the ENs due to reduced return traffic will increase. However, as the number of contents increases, only a portion of them can be cached in the ENs due to the limited cache space, resulting in a decrease in CHR and an increase in content delivery delay. Therefore, the utility obtained by the ENs for delivering contents to UTs in advance will decrease.

When the number of contents increases, the local cache can only meet the demands of some terminals. Some contents cannot be cached in ENs, which own the limited edge cache space; thus, more contents need to be obtained from clouds. Compared to the RCC algorithm and the GCC algorithm, the proposed algorithm considers both the content popularity and the user access delay when caching contents, thus having better performance.

The cost caused by the number of contents will increase with the rising number of contents. The reason is that the contents need to be cached less when the number of contents grows, which will result in the decrease of CHR. Accordingly, the cost increases during the content caching process.

For example, when the number of contents was 500, the CHR of the proposed algorithm was 49.23% higher than that of the RCC algorithm and 9.67% higher than that of the GCC algorithm, the ACRD of the proposed algorithm was 43.11% higher than that of the RCC algorithm and 8.11% higher than that of the GCC algorithm, and the ASU of proposed algorithm improved by up to 34.85% and 5.95% compared to that of the RCC algorithm and the GCC algorithm, respectively.

#### 6.5.3. The Influence of the Cache Space on Metrics

**Figure 10:**
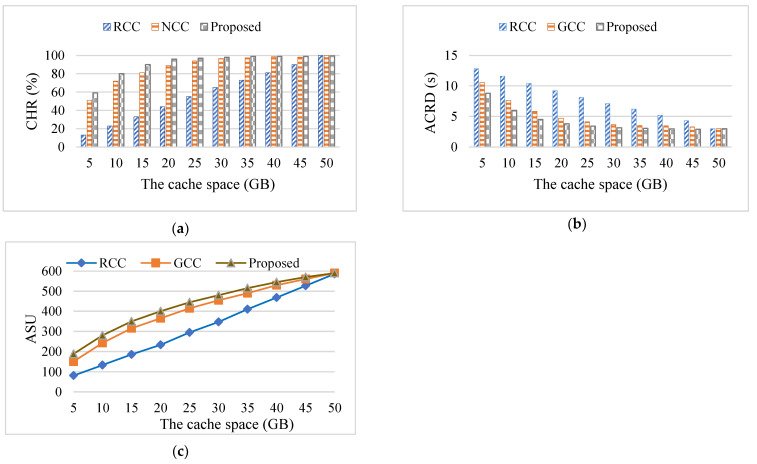
The influence of the cache space on the metrics: (**a**) the influence of the cache space on CHR; (**b**) the influence of the cache space on ACRD; (**c**) the influence of the cache space on ASU.

In order to verify the influence of the cache space on metrics, the Zipf parameter was set to 0.6, the number of contents was set to 1000, and the content size was between 10 MB and 50 MB in the experiment. The number of UTs was set to 20. Keeping other control parameters constant, the cache size of each EN was set to be equal, and the cache capacity was gradually adjusted from 5 GB to 50 GB. Figure 10 shows the influence of the cache space on metrics. The experiment was divided into 10 groups, and each group was conducted 20 times, with the average value used as the final experimental result.

The size of the cache space influenced the cache capability of the edge nodes. The larger cache space resulted in the increase of CHR and ASU and the decrease of ACRD. Figure 10a shows that the value of CHR became large with the increase of cache space. This is because the number of cached contents increased as the cache space of ENs grew, and as the probability that the user requests can be served by ENs increased, the CHR of ENs also increased. Obviously, the CHR of the RCC algorithm increased linearly with the increase in cache space, while the CHR of the GCC algorithm and the proposed algorithm showed more obvious advantages when the cache capacity was insufficient, and their CHR was higher than that of the RCC algorithm. However, with the increase in cache capacity, the CHR of all strategies tended to be to 1. In theory, when the cache capacity is large enough, all contents in the content library can be cached to the ENs so that all user requests can be satisfied, and the CHR of all algorithms tends to be similar.

Figure 10b shows a decline of ACRD with the increase of cache space. For the same cache space, the proposed algorithm had the lowest ACRD. However, when the cache space increased to 50 GB, the ACRD of all algorithms tended to be the same. When the edge cache capacity is large enough, all contents in the content library can be cached to the ENs. In this way, users can download content directly from the local cache without having to retrieve it from the cloud, and the content delivery delay becomes the data transmission delay from the edge node to the intelligent terminal. If the number of user requests remains the same, then the data transmission delay is basically stable.

Figure 10c depicts the rise of ASU with the increase of cache space. The ASU includes the revenue obtained from renting cache space from each EN, the benefit obtained from reducing backhaul traffic, and the reward obtained from delivering content in advance in this paper. The reason for the change trend is that the larger the cache capacity of ENs, the more content they can cache, and the greater the utility that they can obtain by renting cache space. On the other hand, the more content is cached at the edge, the lower the average delivery delay of the content, and the more rewards can be obtained by delivering content in advance. In addition, the more content is cached at the edge, the more backhaul traffic is reduced, and the higher utility obtained from reducing backhaul traffic. The ASU of the proposed algorithm was better than that of the benchmark algorithms.

It is worth mentioning that the edge cache space cannot be deployed very large in reality. This is because physical cache devices require high deployment costs, which are much higher than the maintenance costs of cache space within a single time slot. Therefore, when deploying cache space size, both the cache benefit and deployment cost should be considered, and appropriate cache space should be selected to balance the cache benefit and deployment cost.

On the one hand, the GCC algorithm and the proposed algorithm both consider the popularity of contents and cache popular contents to meet the demands of more UTs. On the other hand, the figures show that the proposed algorithm and the RCC algorithm saved more cache space and saved cache costs when the cache capacity was small. In addition, the proposed algorithm also considers the influence of content size, which can cache more contents in limited space, thus making it slightly superior to the GCC algorithm in terms of CHR and ACRD. Additionally, the proposed algorithm not only considers the utility obtained from renting cache space and saving backhaul traffic, but also considers the mechanism of rewarding for delivering content in advance; thus, the proposed algorithm was able to obtain larger node utility.

When the cache space was 20 GB, the CHR of the algorithm in this paper was 134.15% and 7.87% higher than that of the RCC algorithm and the GCC algorithm, respectively, the ACRD of the proposed algorithm was reduced up to 56.32% compared to the RCC algorithm and up to 19.32% compared to the GCC algorithm, and the ASU of the proposed algorithm was improved by up to 50.85% and 7.23% compared to that of the RCC algorithm and the GCC algorithm, respectively.

#### 6.5.4. The Influence of the Number of UTs on Metrics

**Figure 11:**
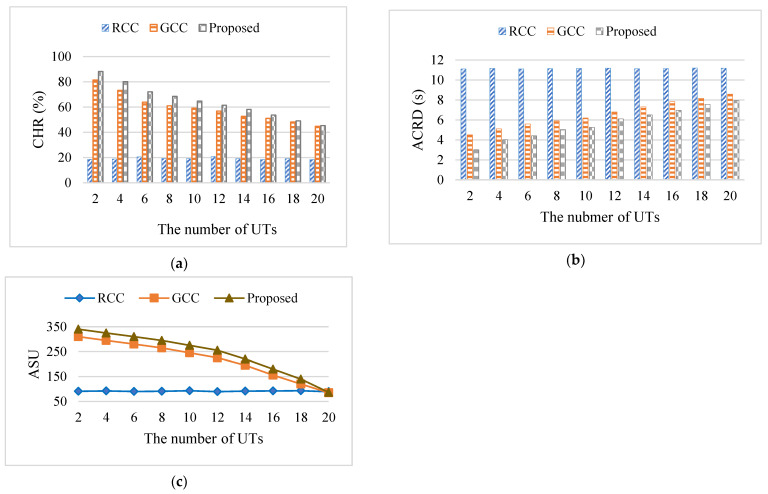
The influence of the number of UTs on the metrics: (**a**) the influence of the number of UTs on CHR; (**b**) the influence of the number of UTs on ACRD; (**c**) the influence of UTs on ASU.

In order to verify the influence of the number of UTs on metrics, the method of controlling variables was used to keep other parameters constant and vary the number of smart terminals from 2 to 20. In this experiment, the Zipf parameter was set to 0.6, the number of contents was set to 1000, and the content size was between 10 and 50 MB. The cache capacity of the ENs was set to 10G. Figure 11 describes the influence of the number of UTs on metrics. The experiment was divided into 10 groups, and each group was conducted 20 times, with the average value taken as the final experimental result.

The number of user terminals affected the diversity of contents, which impacted the distribution of content popularity. When the number of user terminals grew, CHR and ASU decreased while ACRD showed an increasing trend. Figure 11a describes the decline of CHR of the proposed algorithm and the RCC algorithm with the increase of the number of UTs. This is because, as the number of UTs increased, the number and variety of contents requested by UTs also increased, while the cache capacity of ENs was limited and could only cache a certain number of contents. Therefore, as the UT request volume increased, more contents could not be obtained from the edge cache, resulting in a decrease in CHR. The CHR of the RCC algorithm remained at a low level, and its change curve fluctuated within a small range as the number of UTs increased. Due to the limitation of the cache capacity of ENs, the CHR of the proposed algorithm was slightly higher than that of the GCC algorithm.

Figure 11b depicts the change of ACRD with the increasing number of UTs. As the number of UTs increased, the ACRD of the GCC algorithm and the proposed algorithm increased, while the RCC algorithm fluctuated less but had the highest delay. With the limited cache capacity of ENs, the proposed algorithm tended to cache popular content to meet the needs of most UTs. Thus, as the terminals became more and more dense, the delay of the GCC algorithm became closer to that of the proposed algorithm.

Figure 11c shows the decrease of ASU with the increasing number of UTs. The reason is that, as the number of UTs increased, the cost of backhaul traffic for obtaining contents increased due to the decrease in CHR. In addition, the decrease in the reward obtained by ENs for delivering contents in advance to users before the request deadline was due to the increase in average content delivery delay of users. Therefore, the cache utility of ENs decreased. The RCC algorithm had the smallest cache utility, and its ASU level fluctuated slightly as the number of UTs increased. The ASU of the GCC algorithm became closer to that of the proposed algorithm as the number of UTs became more intense, and the ASU of the proposed algorithm was slightly higher than that of the GCC algorithm.

The RCC algorithm did not consider the content popularity when caching and randomly cached content, which could only satisfy the needs of a small number of UTs, while the GCC algorithm and the proposed algorithm tended to cache the more popular content to meet the needs of most UTs, which saved backhaul traffic to some extent and obtained more cache utility. The proposed algorithm also considered the size of the content, adopted incentive measures, and considered the reward for delivering content in advance. Thus, the proposed algorithm can achieve higher performance.

The cost caused by the number of UTs increased with the rising number of UTs. This is because the larger the number of UTs, the more diverse the requested content, and the more dispersed the contents. Thus, the CHR decreases. Accordingly, the cost grows during the process of content caching process.

When the number of UTs was 10, the CHR of the proposed algorithm improved up to 238.39% and 9.56% compared to that of the RCC algorithm and the GCC algorithm, respectively, the ACRD of the proposed algorithm was reduced up to 52.97% and 15.32% compared to that of the RCC algorithm and the GCC algorithm, respectively, and the ASU of the proposed algorithm improved by up to 184.92% and 13.33% compared to that of the RCC algorithm and the GCC algorithm, successively.

In order to verify the performance of the proposed algorithm, the RCC algorithm and the GCC algorithm were taken as the benchmark algorithms. The CHR, the ACRD, and the ASU were taken as the metrics. In the experiments, the influence of the Zipf parameter, the number of contents, the cache space, and the number of UTs on the metrics was discussed, successively. The experimental results show that the proposed algorithm can achieve a better performance regarding the metrics compared to the benchmark algorithms.

## 7. Conclusions

In this paper, the joint optimization strategy of air-ground cooperation caching and content delivery was proposed to reduce the delay of content delivery. Firstly, the content popularity was predicted by the LSTM network based on the time series data of content popularity. Then, the joint optimization problem of air-ground cooperation caching and content delivery based on popularity prediction were built to minimize the total content delivery delay by considering UAV trajectory planning, UAV transmission power allocation, the downlink bandwidth allocation of UAVs and the base station, content caching, and user association. Finally, the block coordinate descent method was adopted to decompose the optimization problem, and the random rounding technique was adopted to restore slack variables to achieve the joint optimization strategy of air-ground cooperation caching and content delivery. The simulation results show that the performance of the proposed algorithm was better than that of benchmark algorithm on average delivery delay, average data transmission energy, and average cache hit rate. In future works, the prototype system of the air-ground cooperation will be built, and the performance of JOA-AGCCCD-PP will be verified in real environments.

## Figures and Tables

**Table 1:** Organization of the existing research.

Ref.	Environment	Strengths	Weaknesses
[6]	A BC-based caching system in the edge and terminal collaborative environment.	Assuring the validity of the content in the system by using a content index method.	Simulation results may not accurately reflect the situation in the real world.
[7]	Edge and terminal collaboration.	Designing an ECS framework for cache resource trading and digital content sharing.	The decentralized framework has limited scalability.
[8]	Edge and terminal collaboration.	Proposing a novel hierarchical architecture of blockchain, which is more suitable for the high-mobility IoV network.	Simulation results may not accurately reflect performance improvements in the real world.
[9]	Cloud-edge-terminal collaboration in the mobile cyber-physical system.	Proposing a novel blockchain-based trustworthy edge caching scheme for mobile users.	The cooperative behaviors of edge nodes need to be addressed.
[10]	Edge and terminal collaboration in a hierarchical wireless network.	Proposing a decentralized framework of proactive caching based on blockchains.	The caching system does not consider the scalability with multiple cache helpers.
[11]	Cloud-edge-end collaborative.	Built the decentralized IoV by combining deep reinforcement learning and the permission BC.	The proposed integration of deep reinforcement learning and blockchain has complexity.
[12]	Cloud-edge-end collaboration in edge-enabled UAV networks.	Proposing a neural BC-based ultrareliable caching.	There are potential challenges in scalability and performance.
[13]	Cloud-edge-terminal collaboration.	Combining edge computing and blockchain to realize efficient authentication and information sharing among IoT platforms.	The proposed system faces the challenge of scalability due to the increase in the number of terminals.
[14]	Cloud-edge-terminal collaborative.	Studied a layered architecture of fog-based IoT applications.	The proposed solutions need to be further validated in a real environment.
[15]	Cloud-edge-terminals collaborative.	Proposing a smart contract framework to ensure security distributed computing in a smart grid.	The proposed scheme can be enhanced further in terms of energy consumption and parallel multi-task scheduling.
[16]	Cloud-edge-terminal collaborative.	Proposing an overview of the issues, challenges, and recommendations of integrated BCT and IoT with DSM and SC.	There is a need for more balanced assessments of BCT benefits and limitations.
[17]	Cloud-edge-terminal collaborative.	Using a case-based reasoning approach to enhance data warehouse performance.	Other AC features need to be combined to make the framework more autonomous.

**Table 2:** The configuration information on hardware.

Node Name	CPU/Memory/Disk	Manufacturer/City/Country
Ali cloud	Intel Core E7-4820/4 GB/500 GB	Intel Group/Santa Clara, CA/USA
EN 1(Lenovo server)	Intel Core i5-4590 CPU/4 GB/2 TB	Intel Group/Santa Clara, CA/USA
EN 2(Lenovo server)	Intel Core i5-2450 MCPU/8 GB/1 TB	Intel Group/Santa Clara, CA/USA
EN 3(Lenovo server)	Intel Core Duo E2160 CPU/4 GB/2 TB	Intel Group/Santa Clara, CA/USA
Huawei P20	Hisilicon Kirin 970/6.00 GB/128 GB	Huawei Group/ Shenzhen/China
Xiaomi 8	Qualcomm SDM845/6.00 GB/128 GB	Qualcomm/San Diego, CA/USA
ThinkPad E450	Intel Core i5-4300U (4 cores with 1.7 GHZ)/8 GB/500 GB	Intel Group/Santa Clara, CA/USA
HP OMEN	Intel i5-7300 HQ (4 cores with 2.5 GHz)/8 GB/1 TB	Intel Group/Santa Clara, CA/USA

**Table 3:** The configuration information on the software.

Software Name	Version	Function
Cloud management tool	OpenStack-Train	Building and managing the cloud resources
Linux operating system	Ubuntu 16.04	The operating system on cloud servers or ENs
Docker	Docker 1.2.16	Building the containers on ENs
K8s	Kubernetes 1.12.8	Managing the edge resources
BC system	Ethereum 1.9.23-stable	Building the BC system and the full nodes
Wallet	Metamask-chrome-10.0.2	Managing the account and creasing the light nodes
IPFS	go-ipfs 0.9.0	Storing the source data

**Table 4:** The values of the parameters.

Parameters	Value
Zipf parameter	[0, 1]
The number of contents	1000
The size of each content	[5, 50] MB
The maximal content delivery delay	[5, 10]s
The caching space of each EN	[5, 50] GB
The number of UTs	[5, 25]

## Data Availability

http://files.grouplens.org/datasets/movielens/ml-latest.zip (accessed on 18 June 2023).

## Abbreviations

DP	The set of DPs
F	The set of contents
$U_{m}$	The set of UTs served by m-th EN
$C_{m}$	The available cache storage size
$D_{q}$	The size of q-th content
$x_{m,q}$	The decision variable of content caching
$p_{q}$	The popularity of q-th content
$prof_{m}$	The node utility
$prof_{m}^{1}$	The edge caching utility
$prof_{m}^{2}$	The content transmission utility
n	The n-th UT
m	The m-th EN
